# Endogenous adenine mediates kidney injury in diabetic models and predicts diabetic kidney disease in patients

**DOI:** 10.1172/JCI170341

**Published:** 2023-10-16

**Authors:** Kumar Sharma, Guanshi Zhang, Jens Hansen, Petter Bjornstad, Hak Joo Lee, Rajasree Menon, Leila Hejazi, Jian-Jun Liu, Anthony Franzone, Helen C. Looker, Byeong Yeob Choi, Roman Fernandez, Manjeri A. Venkatachalam, Luxcia Kugathasan, Vikas S. Sridhar, Loki Natarajan, Jing Zhang, Varun S. Sharma, Brian Kwan, Sushrut S. Waikar, Jonathan Himmelfarb, Katherine R. Tuttle, Bryan Kestenbaum, Tobias Fuhrer, Harold I. Feldman, Ian H. de Boer, Fabio C. Tucci, John Sedor, Hiddo Lambers Heerspink, Jennifer Schaub, Edgar A. Otto, Jeffrey B. Hodgin, Matthias Kretzler, Christopher R. Anderton, Theodore Alexandrov, David Cherney, Su Chi Lim, Robert G. Nelson, Jonathan Gelfond, Ravi Iyengar, for the Kidney Precision Medicine Project

**Affiliations:** 1 Center for Precision Medicine and; 2 Division of Nephrology, Department of Medicine, University of Texas Health Science Center at San Antonio, Texas, USA.; 3 Department of Pharmacological Sciences and Institute for Systems Biomedicine, Icahn School of Medicine at Mount Sinai, New York, New York, USA.; 4 Division of Nephrology, Department of Medicine and Section of Endocrinology, Department of Pediatrics, University of Colorado Anschutz Medical Campus, Aurora, Colorado, USA.; 5 Department of Internal Medicine, University of Michigan, Ann Arbor, Michigan, USA.; 6 SygnaMap Inc., San Antonio, Texas, USA.; 7 Clinical Research Unit, Khoo Teck Puat Hospital, Singapore.; 8 Chronic Kidney Disease Section, National Institute of Diabetes and Digestive and Kidney Diseases, Phoenix, Arizona, USA.; 9 Department of Population Health Sciences and; 10 Department of Pathology, University of Texas Health Science Center at San Antonio, Texas, USA.; 11 Department of Medicine, Division of Nephrology, University Health Network, Toronto, Ontario, Canada. Department of Physiology and Cardiovascular Sciences Collaborative Specialization, University of Toronto, Toronto, Canada.; 12 Herbert Wertheim School of Public Health and; 13 Moores Cancer Center, University of California, San Diego, La Jolla, California, USA.; 14 CeMM Research Center for Molecular Medicine of the Austrian Academy of Sciences, Vienna, Austria.; 15 Department of Health Science, California State University, Long Beach, Long Beach, California, USA.; 16 Section of Nephrology, Department of Medicine, Boston Medical Center and Boston University, Chobanian & Avedisian School of Medicine, Boston, Massachusetts, USA.; 17 Department of Medicine, Division of Nephrology, Kidney Research Institute, University of Washington, Seattle, Washington, USA.; 18 Institute of Molecular Systems Biology, ETH Zurich, Zurich, Switzerland.; 19 Center for Clinical Epidemiology and Biostatistics and Department of Biostatistics, Epidemiology, and Informatics, Perelman School of Medicine at the University of Pennsylvania, Philadelphia, USA.; 20 Patient-Centered Outcomes Research Institute, Washington, DC, USA.; 21 Epigen Biosciences Inc., San Diego, California, USA.; 22 Cleveland Clinic, Cleveland, Ohio, USA.; 23 Department of Clinical Pharmacy and Pharmacology, University Medical Center Groningen, Groningen, Netherlands.; 24 The George Institute for Global Health, Sydney, Australia.; 25 Environmental Molecular Sciences Laboratory, Pacific Northwest National Laboratory, Richland, Washington, USA.; 26 Structural and Computational Biology Unit, European Molecular Biology Laboratory, Heidelberg, Germany.; 27 Diabetes Center, Admiralty Medical Center, Khoo Teck Puat Hospital, Singapore.; 28 Saw Swee Hock School of Public Health, National University of Singapore, Singapore.; 29 Lee Kong Chian School of Medicine, Nanyang Technological University, Singapore.; 30 The Kidney Precision Medicine Project is detailed in Supplemental Acknowledgments.

**Keywords:** Nephrology, Chronic kidney disease, Diabetes, Fibrosis

## Abstract

Diabetic kidney disease (DKD) can lead to end-stage kidney disease (ESKD) and mortality; however, few mechanistic biomarkers are available for high-risk patients, especially those without macroalbuminuria. Urine from participants with diabetes from the Chronic Renal Insufficiency Cohort (CRIC) study, the Singapore Study of Macro-angiopathy and Micro-vascular Reactivity in Type 2 Diabetes (SMART2D), and the American Indian Study determined whether urine adenine/creatinine ratio (UAdCR) could be a mechanistic biomarker for ESKD. ESKD and mortality were associated with the highest UAdCR tertile in the CRIC study and SMART2D. ESKD was associated with the highest UAdCR tertile in patients without macroalbuminuria in the CRIC study, SMART2D, and the American Indian study. Empagliflozin lowered UAdCR in nonmacroalbuminuric participants. Spatial metabolomics localized adenine to kidney pathology, and single-cell transcriptomics identified ribonucleoprotein biogenesis as a top pathway in proximal tubules of patients without macroalbuminuria, implicating mTOR. Adenine stimulated matrix in tubular cells via mTOR and stimulated mTOR in mouse kidneys. A specific inhibitor of adenine production was found to reduce kidney hypertrophy and kidney injury in diabetic mice. We propose that endogenous adenine may be a causative factor in DKD.

## Graphical abstract

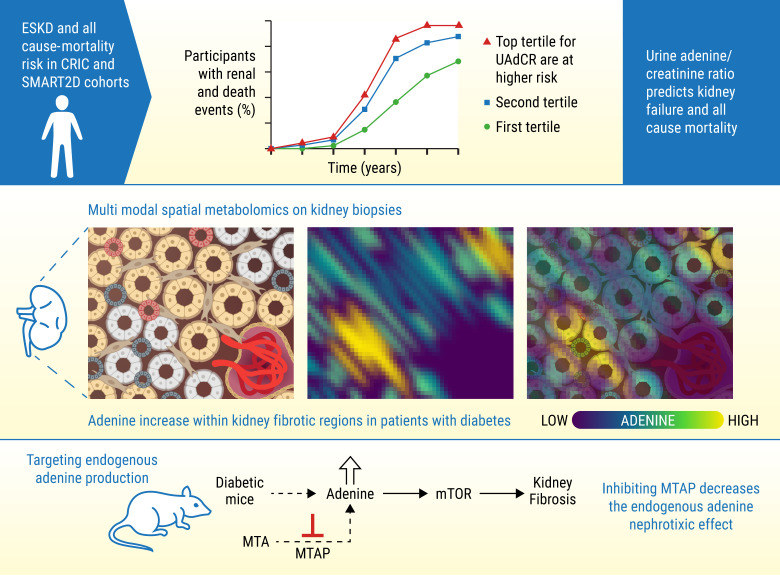

## Introduction

Progression to organ failure is marked by fibrosis and loss of architecture in solid organs, such as the kidney. In almost all progressive chronic kidney diseases (CKDs), the features that are most consistently associated with functional loss of the glomerular filtration rate (GFR) are the degree of glomerulosclerosis, tubulointerstitial fibrosis, vascular injury, and proteinuria (1–4). However, many patients who eventually develop end-stage kidney disease (ESKD) are nonproteinuric at the time impaired GFR is recognized. Nonproteinuria is defined as a urine albumin-to-creatinine ratio (ACR) of less than or equal to 300 mg/creatinine or urine albumin excretion of less than or equal to 300 mg/d (5). As nonproteinuric or nonmacroalbuminuric diabetic kidney disease (DKD) accounts for more than 40% of prevalent ESKD in patients with type 2 diabetes (T2D) (5–7) and 75% of prevalent CKD (GFR, <60 mL/min/1.73 m^2^) (8), identifying the patients at risk for progression in early stages of disease is an important step to improve clinical outcomes. This is especially relevant as the armamentarium of therapies for DKD to mitigate kidney disease progression has rapidly expanded (9–11).

Establishing novel biomarkers that predict progression and represent biologically relevant pathways in DKD could improve the care of patients with diabetes. To identify novel biomarkers, we recently performed an untargeted urine metabolomics study in patients with T2D and impaired estimated GFR (eGFR) from the Chronic Renal Insufficiency Cohort (CRIC) study (12), and we identified 15 candidate metabolites associated with ESKD. A targeted assay validated 13 of these metabolites, one of which was adenine. As exogenous adenine has been found to cause kidney failure in mice, rats, and dogs (13–15), we evaluated whether endogenous adenine could play a role in progression of kidney disease in patients with diabetes.

## Results

### Urine adenine/creatinine ratio predicts kidney failure and all-cause mortality in the CRIC and SMART2D cohorts.

**Figure 1:**
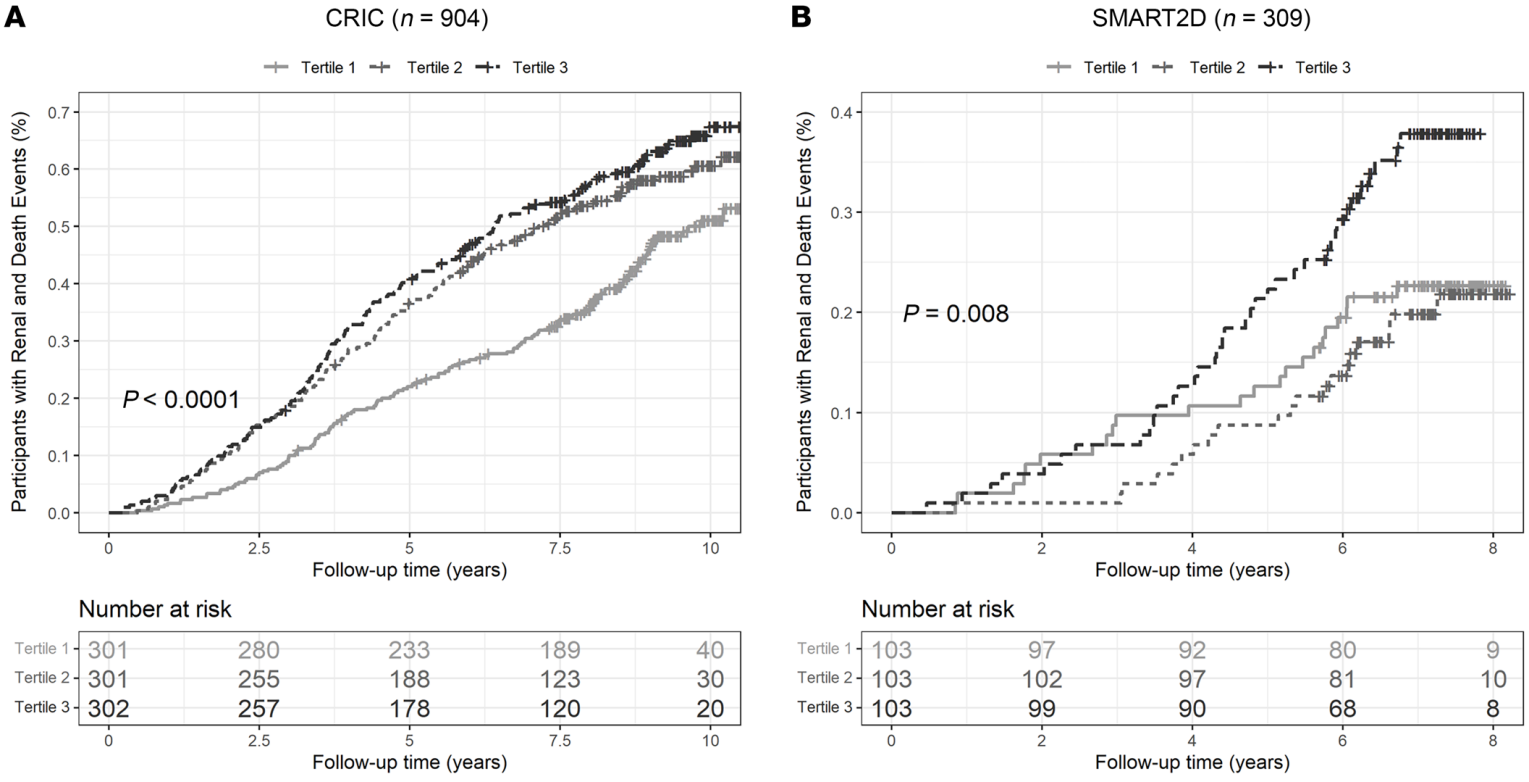
High urine adenine/creatinine levels identify patients with diabetes who are at high risk of end-stage kidney disease and mortality. (**A**) Participants with diabetes in the CRIC cohort (*n* = 904) had urine adenine/creatinine ratios (UAdCRs) measured within 1 year of enrollment and followed for 10 years. The participants in the top tertile had the highest risk of end-stage kidney disease (ESKD) and all-cause mortality. (**B**) Participants from the SMART2D study (*n* = 309) had UAdCR measurements at the time of enrollment and were followed for 7 years. The participants in the top tertile for UAdCR had the highest risk for ESKD and all-cause mortality. A log-rank test was used to compare cumulative incidence curves in **A** and **B**. A *P* value of less than 0.05 was considered significant.

The baseline clinical characteristics of the participants with diabetes from the CRIC and Singapore Study of Macro-angiopathy and Micro-vascular Reactivity in Type 2 Diabetes (SMART2D) study are shown in Table 1. Of the 904 participants evaluated from the CRIC study, 558 had either normoalbuminuria or microalbuminuria, 341 had macroalbuminuria, and 5 had no data for 24-hour albumin. The mean eGFR was 40 mL/min/1.73 m^2^. The top tertile of baseline urine adenine/creatinine ratio (UAdCR) was found to identify the participants with diabetes who were at high risk for ESKD and all-cause mortality (adjusted HR, 1.57; 95% CI, 1.18–2.10; as compared with the lowest tertile) (Figure 1A), and a similar significant relationship was found using UAdCR as a continuous variable (Table 2). The top tertile of UAdCR was valuable as a tool to identify patients with diabetes at high risk for ESKD and all-cause mortality was confirmed in participants from the SMART2D study who had reduced eGFR and normoalbuminuria or microalbuminuria (adjusted HR, 1.77; 95% CI, 1.00–3.12) (Figure 1B and Table 2, data sets combined in Supplemental Figure 2A; supplemental material available online with this article; https://doi.org/10.1172/JCI170341DS1).

### UAdCR predicts kidney failure in the nonmacroalbuminuric American, CRIC, and SMART2D cohorts and empagliflozin reduces UAdCR.

**Figure 2:**
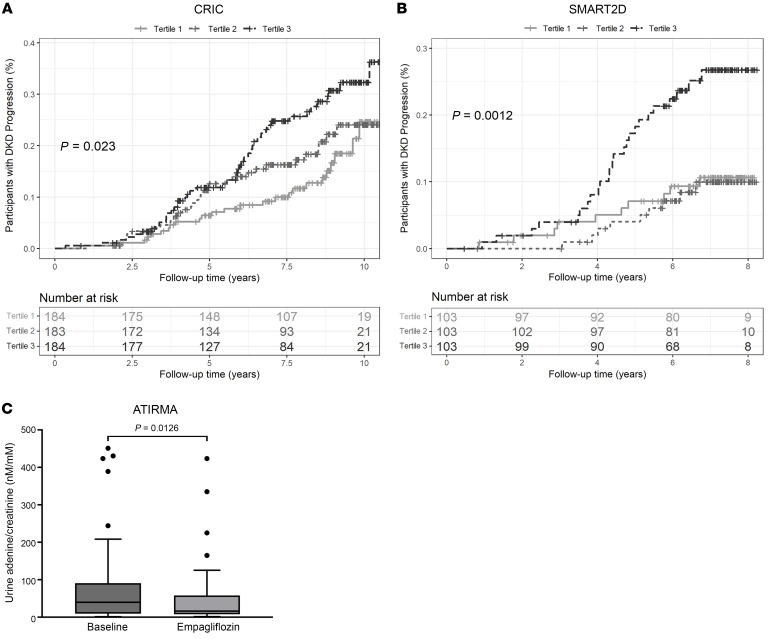
High urine adenine/creatinine tertile identifies end-stage kidney disease outcome in nonmacroalbuminuric patients with diabetes and empagliflozin-reduced urine adenine/creatinine ratio. (**A** and **B**) The participants with the top urine adenine/creatinine ratio (UAdCR) tertile had a significant increase in risk of end-stage kidney disease (ESKD) from the (**A**) CRIC (*n* = 551) and (**B**) SMART2D (*n* = 309) studies. (**C**) Patients with T1 diabetes underwent treatment with empagliflozin for 8 weeks, which reduced UAdCR levels (*n* = 40 patients). A log-rank test was used to compare cumulative incidence curves in **A** and **B**, and a 2-sample *t* test was used to compare UAdCR levels in **C**. A *P* value of less than 0.05 was considered significant.

The UAdCR was also evaluated in early-stage disease (measured GFR >90 mL/min/1.73 m^2^) in a American Indian cohort with more than a 20-year follow-up (Table 1). As the majority of the participants in the American Indian cohort had nonmacroalbuminuria (*n* = 42 of the 54 participants), the association of UAdCR with longitudinal progression to ESKD was presented in this nonmacroalbuminuric cohort. ESKD was associated with the top UAdCR tertile (HR, 4.47; 95% CI, 1.53–13.06) (Supplemental Table 1). UAdCR was also measured in 2 untimed spot urine samples obtained 1 year apart, and it was found to be consistent across the individual paired samples (*r* = 0.665, *P* < 0.0001) (Supplemental Figure 3). Similar relationships that could be used to predict ESKD were found in the nonmacroalbuminuric participants in the CRIC study (adjusted HR, 2.36; 95% CI, 1.26–4.39) and SMART2D (adjusted HR, 2.39; 95% CI, 1.08–5.29) (Figure 2, A and B, and Supplemental Table 2, combined data sets are shown in Supplemental Figure 2B). Of note, there were no significant correlations of UAdCR with the UACR or eGFR in the nonmacroalbuminuric participants from the CRIC study or SMART2D (Supplemental Table 3). Of the CRIC study participants with macroalbuminuria, there were modest associations between the top tertile of UAdCR and ESKD (HR, 1.10; 95% CI, 0.75–1.60) and mortality (HR, 1.33; 95% CI, 0.59–3.01).

To determine if UAdCR could be modified in nonmacroalbuminuric participants with normal or elevated measured GFR by glycemia or a therapeutic intervention with an SGLT2 inhibitor, the UAdCR was measured during euglycemia or hyperglycemia before and after empagliflozin in patients with T1D (clinical characteristics described in Supplemental Table 4). Acute hyperglycemia did not alter UAdCR levels (Supplemental Figure 4); however, empagliflozin significantly lowered UAdCR by 36.4% (Figure 2C).

### Adenine is localized to regions of kidney fibrosis and is increased in patients with diabetes.

**Figure 3:**
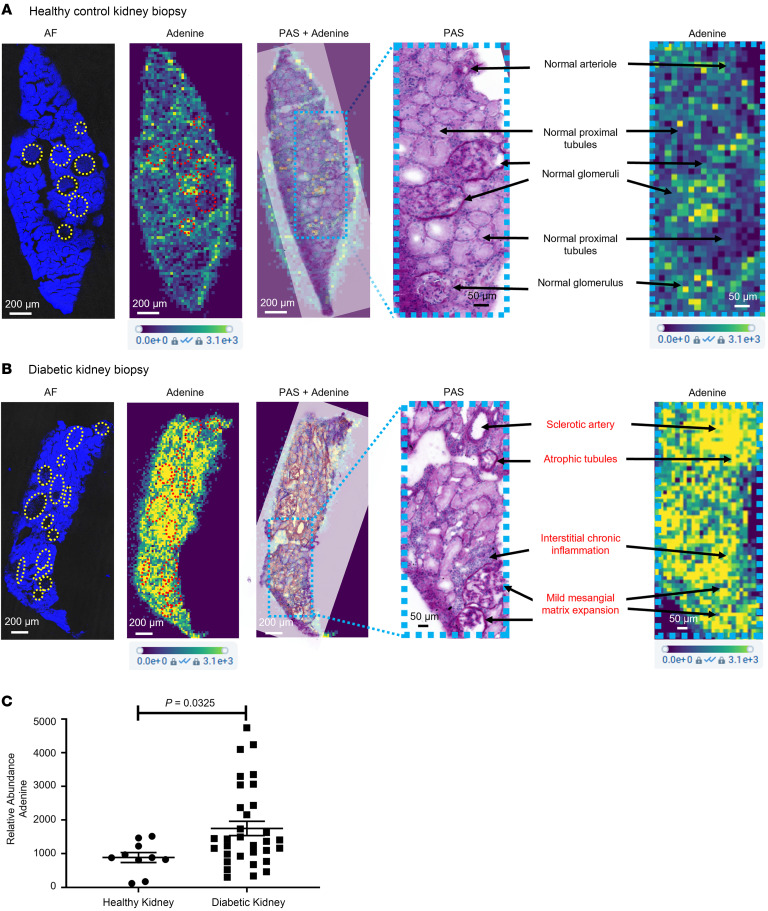
Spatial metabolomics identifies adenine in regions of pathology in nonmacroalbuminuric patients with diabetes. (**A**) Adenine was localized to regions of normal glomeruli and vessels in the normal kidney. Yellow circles and red circles indicate the region of interest labeled on the AF image and adenine ion image, respectively. AF, autofluorescence. Scale bar: 200 μm (AF, adenine, PAS + adenine, and adenine); 50 μm (PAS). (**B**) In a diabetic kidney, adenine is diffusely increased across the tissue section and prominent in regions of sclerotic blood vessels, glomeruli with mild sclerosis, and regions of atrophic tubules and interstitial inflammation. Scale bar: 200 μm (AF, adenine, PAS + adenine, and adenine); 50 μm (PAS). (**C**) Quantitative assessment across healthy controls (*n* = 5 from the CROCODILE study) and diabetic samples (*n* = 8 T1D from CROCODILE and *n* = 8 T2D, 2 from CROCODILE and 6 from Kidney KPMP) demonstrates a statistically significant increase of adenine in kidney tissue sections. Two-tailed Student’s *t* test was used for the comparison. Data represent mean ± SEM.

A spatial metabolomics platform was developed to annotate small molecules (<700 Da) and performed on kidney biopsies from individuals acting as healthy controls and patients with diabetes (clinical characteristics in Supplemental Table 4). Adenine was present at low intensity in normal glomeruli and blood vessels in the healthy control kidney (Figure 3A) and enhanced in regions of arteriolosclerosis, tubulointerstitial fibrosis, and early glomerulosclerosis in the diabetic kidney (Figure 3B). There was an overall increase in adenine in the whole section of kidney biopsies from participants with diabetes as compared with those from individuals acting as healthy controls (Figure 3C). The spatial adenine values in rat kidney sections were found to correlate well with the UAdCR in a Zucker diabetic fatty (ZDF) diabetic model (*r* = 0.73, *P* < 0.001; Supplemental Table 5).

### Single-cell transcriptomics identify ribonucleoprotein biogenesis as a dominant pathway in nonmacroalbuminuric DKD.

**Figure 4:**
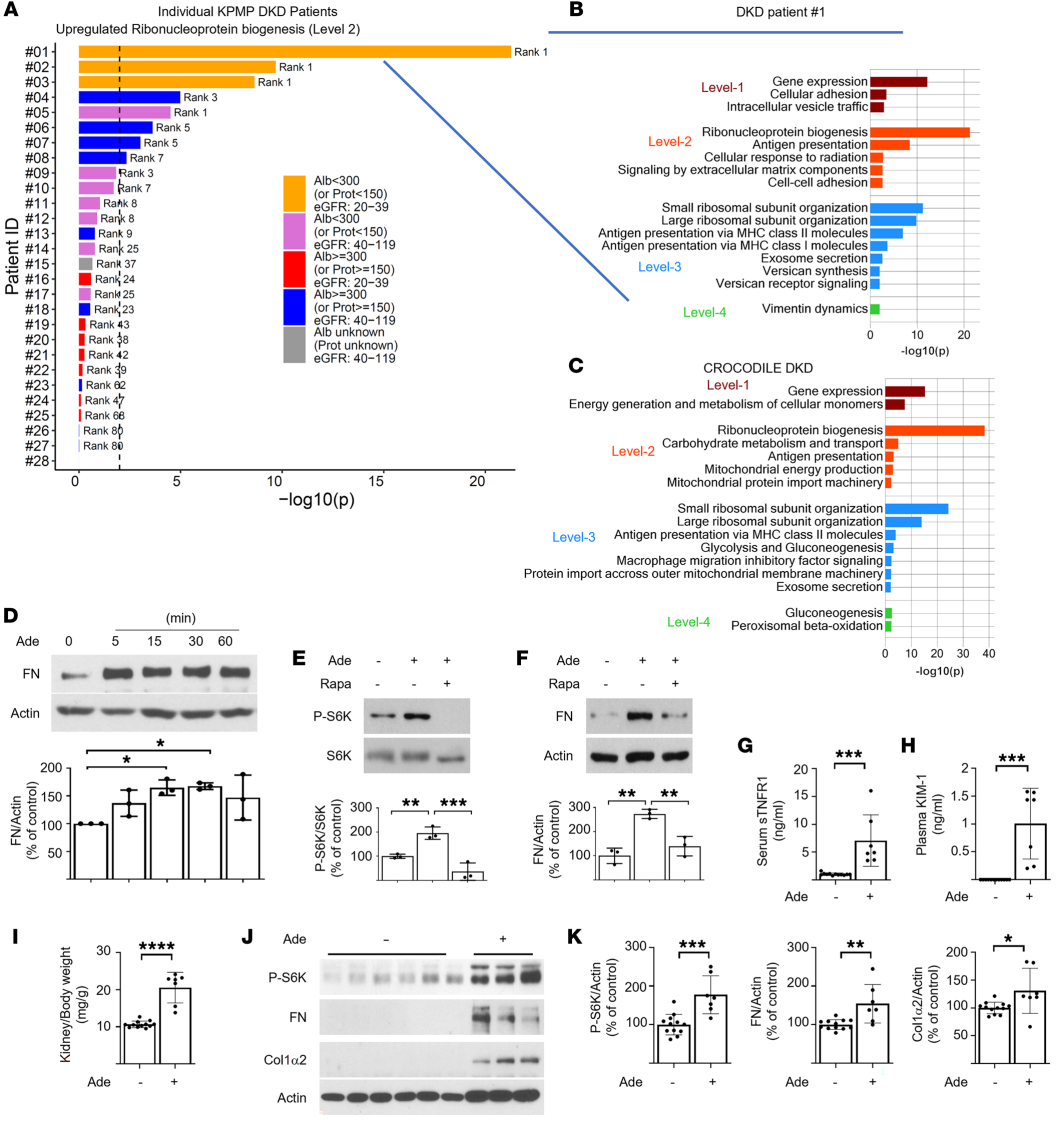
Molecular pathways and events implicating the ribonucleoprotein biogenesis and mTOR pathways with adenine in DKD. (**A** and **B**) The protein synthesis (ribonucleoprotein [RNP] biogenesis) pathway increased in proximal tubule cells of patients with DKD without proteinuria. Single-cell- transcriptomic data obtained from DKD kidney biopsies from the KPMP study were analyzed for differentially expressed genes in proximal tubules (PTs) of each DKD patient versus healthy reference tissue. Upregulated genes with an adjusted *P* ≤ 0.01 and ranked among the top 600 significant differentially expressed genes were subjected to pathway enrichment analysis using the Molecular Biology of the Cell Ontology (MBCO). Ranking for the RNP biogenesis pathway (a level-2 pathway canonically regulated by the mTOR pathway) is shown for 28 individual patients. Vertical dashed lines indicate *P* ≤ 0.01 for pathway ranking. (**C**) Up to the top 5, 5, 10, and 5 level-1 (dark red), level-2 (red), level-3 (blue), and level-4 (green) pathways, respectively, using MBCO are shown for patient 1 (*P* ≤ 0.01). See blue lines in **A** and **B**. Single-cell-transcriptomic data from patients with T2D (*n* = 6) with low albuminuria compared with cohort specific healthy samples was analyzed to identify upregulated pathways in PT cells. Note that the RNP biogenesis pathway is the top ranked level-2 pathway in both independent studies. (**D**–**F**) Cell culture studies in mouse proximal tubular cells demonstrated an increase in (**D**) fibronectin and (**E**) phospho-S6 kinase and (**F**) that mediation of fibronectin (FN) upregulation is blocked by rapamycin, indicating that mTOR mediates adenine effect. (**G**–**K**) Adenine administration to mice increases (**G**) serum soluble tumor necrosis factor-1 (sTNFR1) and (**H**) plasma kidney injury marker-1 (KIM-1) and (**I**) stimulates kidney and (**J** and **K**) matrix molecules in the kidney (*n* = 12 in control group and *n* = 7 in adenine treated group). *P < 0.05, **P < 0.01, ***P < 0.001, ****P < 0.0001, 2-tailed Student’s *t* test was used for 2 group comparisons. One-way ANOVA was used for multiple group comparisons. Data represent mean ± SD.

As adenine was prominent in regions of tubular pathology in the diabetic kidney and empagliflozin treatment lowered the UAdCR in patients, proximal tubular cells were considered to be a target cell type affected by adenine. Single-cell transcriptomics of proximal tubular cells from patients with DKD from the Kidney Precision Medicine Project (KPMP) study (*n* = 28) and an unbiased pathway analysis were performed based on differentially regulated genes. The top pathway identified was the ribosomal nucleoprotein biogenesis pathway in patients without macroalbuminuria and low eGFR (Figure 4, A and B). In addition, small and large ribosomal subunit organization pathways were also upregulated in these patients. Replication of these results from the KPMP study was found in the Control of Renal Oxygen Consumption, Mitochondrial Dysfunction, and Insulin Resistance (CROCODILE) study in patients with diabetes without macroalbuminuria and normal GFR (Figure 4C). As ribonucleoprotein biogenesis and small and large ribosomal subunit organization is closely linked to activity of mTOR (16), and adenine has been found to stimulate mTOR (17), this pathway was evaluated for its ability to mediate adenine-induced effects on proximal tubular cells.

### Mechanism of adenine-induced matrix production is via the mTOR pathway, and adenine increases KIM1 and sTNFR1 in mice.

To determine whether adenine could be in the causative pathway for tissue fibrosis, adenine was added to mouse and human proximal tubular cells. There was a robust and early stimulation of fibronectin by adenine (Figure 4D and Supplemental Figure 5A). In addition, adenine stimulated mTOR activity, as demonstrated by enhanced phosphorylation of S6 kinase (Figure 4E and Supplemental Figure 5B). Inhibition of mTORC1 with rapamycin blocked adenine-induced production of fibronectin (Figure 4, E and F). Exposure of adenine to normal mice stimulated blood and kidney levels of soluble tumor necrosis factor receptor 1 (sTNFR1) and kidney injury molecule-1 (KIM1), kidney hypertrophy, kidney mTOR activity, and kidney matrix production (Figure 4, G–K, and Supplemental Figure 6).

### Endogenous adenine contributes to DKD in db/db mice.

**Figure 5:**
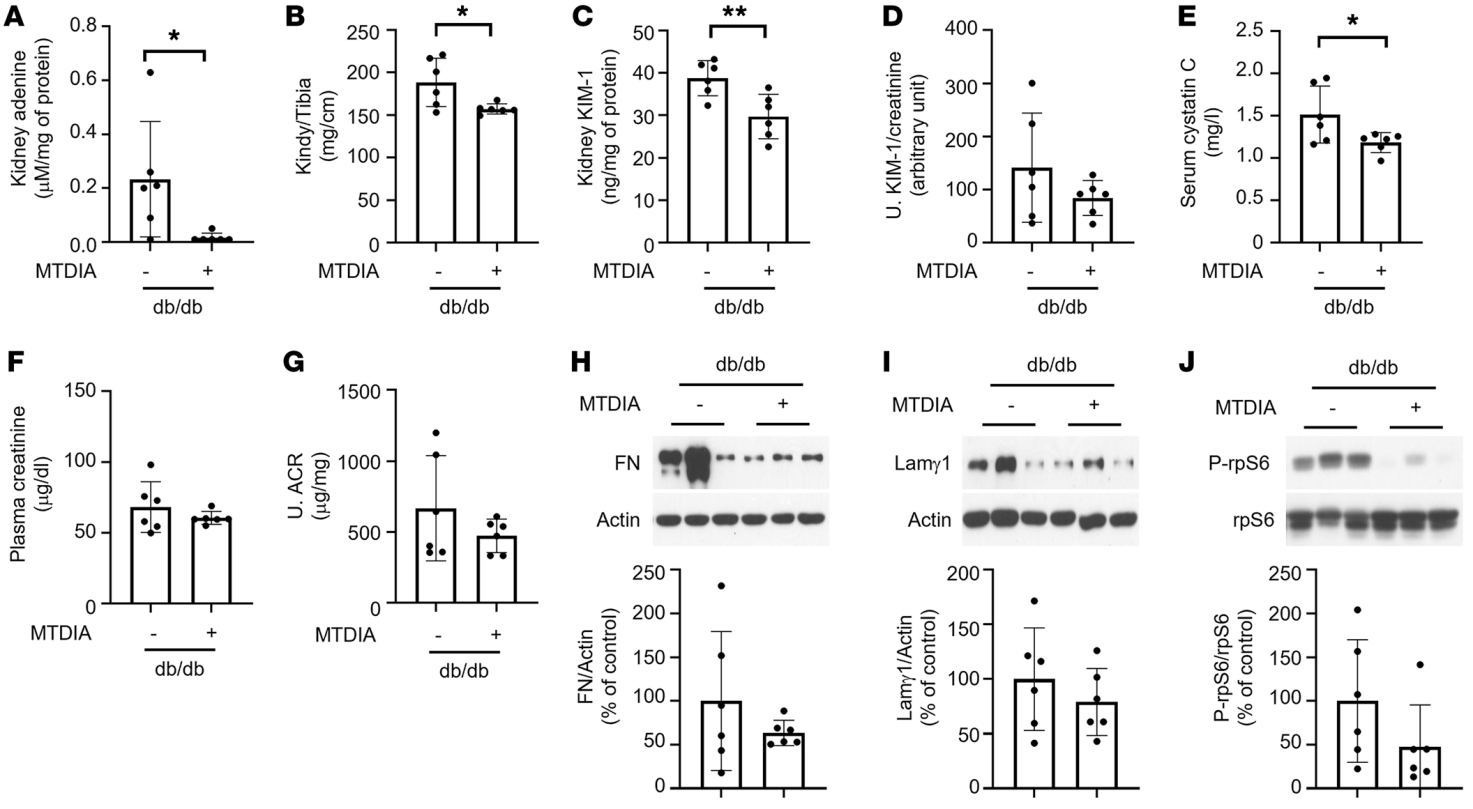
Methylthioadenosine phosphorylase inhibitor ameliorates kidney injury in db/db mice with type 2 diabetes. (**A**–**C**) Methylthio-DADMe-Immucillin-A (MTDIA) significantly reduced kidney (**A**) adenine levels, (**B**) kidney hypertrophy, (**C**) kidney KIM-1 levels, and (**D**) urine KIM-1 levels in diabetic mice. (**E**–**G**) MTDIA significantly reduced (**E**) diabetes-increased serum cystatin C and (**F**) partially reduced plasma creatinine and (**G**) albuminuria in diabetic mice. (**H** and **I**) Diabetes-induced kidney matrix protein levels were partially reduced by MTDIA. (**J**) Ribosomal S6 phosphorylation was partially reduced by MTDIA in the kidneys of db/db mice (*n* = 6 per group). *P < 0.05, **P < 0.01, ***P < 0.001, 2-tailed Student’s *t* test was used for 2 group comparisons. Data represent mean ± SD.

To determine whether endogenous adenine plays a role in progression of DKD, methylthio-DADMe-Immucillin-A (MTDIA), a small-molecule-specific inhibitor of methylthioadenosine phosphorylase (MTAP), was administered to db/db mice, a model of obese T2D. MTAP converts methylthioadenosine to adenine and is responsible for approximately 80% of adenine production in mammalian cells (18). MTDIA was well tolerated and did not affect food intake, water intake, blood glucose levels, or body weight (Supplemental Table 6). MTDIA significantly reduced kidney adenine in db/db mice (Figure 5A) but not other metabolites linked to progression of kidney disease (Supplemental Table 7) (12). MTDIA significantly reduced serum cystatin C, kidney hypertrophy, and kidney KIM1 and partially reduced urine ACR, serum creatinine, urine KIM1, kidney matrix proteins, and mTOR activity in db/db mice (Figure 5, B–J).

## Discussion

The results from this study demonstrate a role for endogenous adenine in kidney disease progression in the context of DKD. Urine levels of the AdCR identified patients with diabetes at high risk of kidney failure and all-cause mortality at all levels of albuminuria in the CRIC study, and this was verified in a cohort study from Singapore. The UAdCR could also identify patients who will develop ESKD even in the setting of normal or elevated GFR without macroalbuminuria across ethnicities. Spatial metabolomics localized adenine to regions of vascular, tubular, and glomerular pathology in patients with diabetes who have normoalbuminuria and GFR. Adenine appears to be in the causal pathway of kidney fibrosis, as adenine was demonstrated to stimulate matrix molecules in proximal tubular cells via mTOR and was causative of kidney matrix production in mice, and inhibiting adenine production was protective in diabetic mice.

Biomarkers in the potentially causal pathways have not previously been identified for kidney disease progression in nonmacroalbuminuric patients with diabetes to our knowledge. Microalbuminuria is clearly a risk factor for kidney disease progression; however, as microalbuminuria can revert to normoalbuminuria (19) the dependence upon microalbuminuria alone may not provide reliable prognostication for event rates of GFR decline or kidney failure. Noninvasive omics approaches using plasma and urine have identified promising candidate biomarkers (20–22); however, demonstration of a contributory role of these biomarkers to the disease process has been difficult to establish (23). In the present study, integration of spatial metabolomics and single-cell transcriptomics of human kidney biopsies converged on adenine and the mTOR pathway as highly relevant to DKD progression. The link between adenine and pathologic features of DKD progression was suggested by spatial metabolomics, as adenine could be localized adjacent to atrophic tubules and in regions of arteriosclerotic blood vessels and glomerulosclerosis. The spatial localization implicated adenine as a potential endogenous profibrotic factor.

Adenine is known to cause kidney pathology as an exogenous toxin in mouse (24) and rat models (14) of CKD and possibly as an endogenous toxin in humans (25). The pathology of adenine-induced kidney disease includes glomerulosclerosis, tubular atrophy, interstitial fibrosis, and inflammatory cell infiltration (26, 27). The mechanism of adenine-induced kidney disease has not been established, although it has been postulated that conversion of adenine to 2,8-dihydroxyadenine (25) is a driver of CKD in patients with mutations of adenine phosphoribosyltransferase (APRT), the major enzyme that metabolizes adenine to AMP. However, patients with CKD with APRT mutations are rare. Adenine itself is likely an endogenous tubular toxin based on the spatial metabolomic analysis and our finding that high urine adenine identified patients at high risk of ESKD. Adenine exposure enhances tubular cell matrix production via the mTOR pathway, and a prior study found that adenine is a potent stimulus for mTOR (17). Several published studies in mice and rats have also found that inhibiting mTOR protects against adenine-induced kidney disease (28–30). The mTOR pathway is likely relevant to human DKD, as a recent study found stimulation of mTOR activity in kidney biopsies from patients with DKD (31) and our study with kidney biopsies from KPMP and CROCODILE demonstrated that a number of outputs of mTOR are elevated in DKD. This includes pathways involved in bioenergetics and pathways related to stimulation of extracellular matrix molecules. Furthermore, adenine can increase the levels of KIM1 and sTNFR1, demonstrating that adenine is likely an initiator of downstream injury and inflammatory markers. Endogenous adenine production was blocked with a specific small-molecule inhibitor of MTAP (MTDIA) and found to protect against diabetic renal hypertrophy and elevation of kidney KIM1 and was protective of decline in kidney function, as measured by serum cystatin C. It is possible that chronic MTAP inhibition with MTDIA could be developed as a safe therapeutic, as a prior study found that MTDIA extended life span in mice with colon cancer, and it was provided for 294 days without evidence of toxicity (32). The role of adenine to accentuate mortality is not clear, although it is possible that adenine could be directly toxic to vascular cells.

The UAdCR measurement was closely associated with DKD progression in the nonmacroalbuminuric diabetic American Indian, the CRIC study, and SMART2D cohorts. As nonmacroalbuminuric DKD leads to ESKD in many patients with CKD and diabetes (7, 8, 33), the potentially new UAdCR biomarker could be of clinical value to identify those patients likely to progress. Furthermore, the benefit of SGLT2 inhibitors may be due in part to reduce adenine levels, as our study documented that short term use of empagliflozin significantly attenuated the UAdCR.

Strengths of our study included multiple analysis of several independent cohorts across different ages, ethnicities, and stages of DKD. Additional strengths include application of spatial metabolomics and single-cell transcriptomics to identify a pathway linking adenine to mTOR in human kidney disease pathology and progression. A limitations of our study is that the role of adenine was not demonstrated in type 1 DKD and other causes of CKD.

In conclusion, urine samples from independent well-characterized cohorts of patients with diabetes identified the UAdCR as a robust predictor of ESKD and mortality independent of albuminuria and baseline eGFR, and spatial metabolomic and single-cell-transcriptomic studies from human kidney biopsies identified a potential role for endogenous adenine and the mTOR pathway in DKD. Studies in cells and mice identified a causative role for adenine, and a small-molecule therapeutic was found to block adenine production and was nephroprotective in a mouse model of T2D. Our results thus demonstrate that endogenous adenine could contribute to progressive kidney disease in the context of T2D.

## Methods

### Clinical cohorts.

The parent CRIC study recruited a racially diverse group aged 21–74 years; approximately 50% of recruits had diabetes, with a broad range of kidney function (34). The current study analyzed urine samples at study entry (from baseline 24-hour urine samples) from 904 CRIC study participants with diabetes and eGFR between 20 and 70 mL/min/1.73 m^2^ and sample and outcome data available. SMART2D is an ongoing prospective cohort study of Southeast Asian participants with T2D recruited between 2011 and 2014 (35). Fasting spot urine samples were collected at baseline and stored at –80°C. To validate findings from the CRIC cohort, 309 participants from the SMART2D cohort with baseline eGFR of 20–70 mL/min/1.73 m^2^ and urine ACR of less than 300 μg/mg were evaluated. American Indians with early DKD were enrolled in a randomized clinical trial (36) (ClinicalTrials.gov NCT00340678). GFR was measured annually throughout the trial by the urinary clearance of iothalamate. Stored spot urine samples collected for 2 consecutive years were available from 54 participants and included for analysis. Additionally, urine samples were obtained under controlled euglycemic and hyperglycemic clamp conditions from a previously published clinical study in patients with T1D without macroalbuminuria (*n* = 42) to evaluate the effects of empagliflozin (Adjunctive-to-insulin and Renal Mechanistic [ATIRMA], NCT01392560) (37). Euglycemic clamp (4–6 mM glucose) conditions were maintained for approximately 4 hours before urine collection. The following day hyperglycemia (9–11 mM glucose) was maintained for 4 hours. Urine samples for adenine measurements were performed on samples obtained at the 4-hour time point following euglycemia or hyperglycemia before and after empagliflozin (25 mg/d) treatment for 8 weeks.

### Urine metabolomics (Zip-Chip analysis).

Urine samples from the American Indian, CRIC, SMART2D cohorts and ATIRMA urines were all analyzed using Zip-Chip (908 Devices) coupled with mass spectrometry (38). A rapid throughput urine adenine/creatinine assay was developed that showed excellent correlation with the gold standard assay using LC-MS/MS (Supplemental Figure 1). The reportable linear range for urine adenine assay was 100 nM to 100 μM, with a limit of detection at 10 nM and coefficient of variation of less than 10% across the reportable linear range. Metabolite separation was achieved with a microfluidic chip that integrates capillary electrophoresis with nanoelectrospray ionization through a Zip-Chip interface. Data acquisition was performed with a Q-Exactive mass spectrometer (Thermo Fisher Scientific) and Xcalibur-Quan Browser software (Thermo Fisher Scientific) for data processing. Detailed procedures were previously published (38).

### Human kidney biopsies.

Human kidney samples were obtained via the KPMP (ClinicalTrials.gov NCT04334707) and the CROCODILE studies (39–41). Samples were frozen in liquid nitrogen and stored at –80 °C until analysis. Snap-frozen sample preparation and sectioning procedures for MALDI–mass spectrometry imaging (MALDI-MSI) were published in ref. 42.

### Animal studies.

ZDF rat kidney and urine samples were provided by Epigen Inc. to verify that kidney spatial adenine correlated with the targeted urine adenine assay. C57BL/6J, db/m, and db/db mice were obtained from The Jackson Laboratory. C57BL/6J mice were administered adenine for 4 weeks in drinking water before sacrifice, and tissues and blood samples were harvested at University of Texas Health Science Center at San Antonio. db/m and db/db mice were administered vehicle or MTDIA MTAP inhibitor for a period of 8 weeks from week 10 to week 18. An Albumin ELISA kit (catalog E101 and E90-134, Bethyl Laboratories Inc.) and creatinine colorimetric kit (catalog ADI-907-030A, Enzo Life Sciences Inc.) were used for the urinary ACR. Serum cystatin C was measured by the Quantikine ELISA kit (catalog MSCTC0, R&D Systems). Plasma creatinine and metabolites in kidney tissue were measured by Zip-Chip-mass spectrometry as previously described (12). Urine and kidney KIM-1 was measured by ELISA (catalog DY1817, R&D System).

### MSI and optical imaging of kidney biopsies.

A multimodal imaging approach was developed to investigate regional localization of metabolites in kidney sections. Bright-field and autofluorescence microscopy outlined glomeruli and tubules, and PAS-H staining revealed regions of pathology in serial sections. MALDI-MSI was performed with a Thermo Fisher Scientific Q Exactive HF-X hybrid quadrupole-Orbitrap mass spectrometer in combination with a novel elevated pressure MALDI/ESI interface (Spectroglyph LLC) (43). Metabolite annotation was performed on METASPACE (44), and the optical image was uploaded to METASPACE and SCiLS Lab for visual overlay of metabolites with optical images to provide an assessment of metabolites associated with normal-appearing and pathologic features. Detailed procedures for MALDI-MSI are available in ref. 45.

### Cell culture.

Human kidney proximal tubular (HK-2) cells were purchased from the ATCC and were cultured as previously described (46). Murine kidney proximal tubular epithelial (MCT) cells were cultured as previously described (47). Cells were treated with 20 μM adenine for the indicated time points with and without rapamycin (Fisher Scientific). Phosphorylation of S6 kinase and ribosomal protein S6 and expression of fibronectin and type 1 collagen α2 were analyzed by immunoblotting using antibodies against phosphor-Thr389-S6 kinase (catalog 9205, Cell Signaling Technology), phosphor-Ser240/244 ribosomal protein S6 (catalog 2215, Cell Signaling Technology), ribosomal protein S6 (catalog 2217, Cell Signaling Technology), MTAP (catalog 62765, Cell Signaling Technology), fibronectin (catalog ab2413, Abcam Plc), type 1 collagen α2 (catalog 14695-1-AP, Proteintech Group Inc.), and β-actin (catalog A2066, Millipore Sigma).

### Bioinformatic and systems medicine analysis.

Single-cell transcriptomics and spatial metabolomics data sets generated from healthy kidney tissue, unaffected tissue in kidney nephrectomy, and biopsy samples (KPMP and CROCODILE) were analyzed as recently described (39–41). The top pathway genes and proteins from the top 600 significant genes or proteins from proximal tubular cells were mapped onto pathways for subcellular processes using the Molecular Biology of the Cell Ontology ontology (48).

### Statistics.

A composite kidney endpoint was defined as sustained kidney replacement therapy, progression to GFR or eGFR of less than 15 mL/min/1.73 m^2^, or more than 50% GFR or eGFR decline from baseline level. All-cause mortality included death from any cause before reaching the ESKD endpoint. Urine adenine was normalized to urine creatinine concentrations and log_2_ transformed. In the CRIC and SMART2D cohorts, the association of urine adenine levels (tertile) with clinical endpoints was studied by multivariable Cox proportional hazard regression models with adjustment for age, sex, ethnicity and race, body mass index, hemoglobin A1c, mean arterial pressure, baseline eGFR, and urine ACR (natural log transformed) as covariates. The group with a UAdCR in the lowest tertile was used as reference. Due to the limited number of cases in the American Indian cohort, we only reported univariate Cox proportional hazards analysis for this cohort. To evaluate the pretreatment and posttreatment effect of empagliflozin on urine adenine in the ATIRMA cohort, we performed a linear regression analysis for repeated measures. Two-tailed Student’s *t* test was used for comparisons of features between 2 groups. A *P* value of less than 0.05 was considered significant.

### Study approval.

For the CRIC study, informed consent was obtained from participants. Protocols were approved by IRBs and Scientific and Data Coordinating Center (Philadelphia, Pennsylvania, USA). The SMART2D study was approved by the Singapore National Healthcare Group, and all participants provided written informed consent. The KPMP and CROCODILE studies were approved by the Institutional Review Board at Washington University, St. Louis, Missouri, and the University of Colorado, respectively, and written consent was obtained from all patients. The American Indian study was approved by the Institutional Review Board of the National Institute of Diabetes and Digestive and Kidney Diseases, and all participants gave written informed consent. Mouse studies were carried out after IACUC approval from the University of Texas Health Science Center at San Antonio.

### Data availability.

Values for all data points in graphs are reported in the Supporting Data Values file. Raw human data used in this study are confidential. Deidentified data are provided in the Supplemental Data file and are available from the corresponding author upon request.

## Author contributions

The first 9 coauthors each made unique and critical contributions to this manuscript, and authorship order was determined after discussion among writing group members. KS, PB, SCL, JJL, RI, RGN, MK, and GZ designed the study. KS, PB, SCL, DC, and RGN acquired funding for the study. GZ, J Hansen, HJL, RM, PB, JJL, HLH, LH, AF, PB, MAV, LK, and FCT acquired or generated the data. GZ, J Hansen, HJL, JJL, HLH, VS Sridhar, BYC, RF, MAV, LK, JBH, and JG analyzed the data. KS, GZ, PB, KRT, MK, RGN, and RI wrote the manuscript. KS, PB, MK, SCL, LN, JZ, VS Sharma, B Kwan, SSW, J Himmelfarb, KRT, B Kestenbaum, TF, HIF, IHB, J Sedor, HCL, J Schaub, RM, EAO, CRA, TA, SCL, RGN, JG, and RI provided scientific guidance and insights. All authors reviewed, edited, and approved the manuscript.

## Supplementary Material

Supplemental data

Supplemental Data File 1 (KPMP Consortium Members)

Supporting data values

## Figures and Tables

**Table 2:**
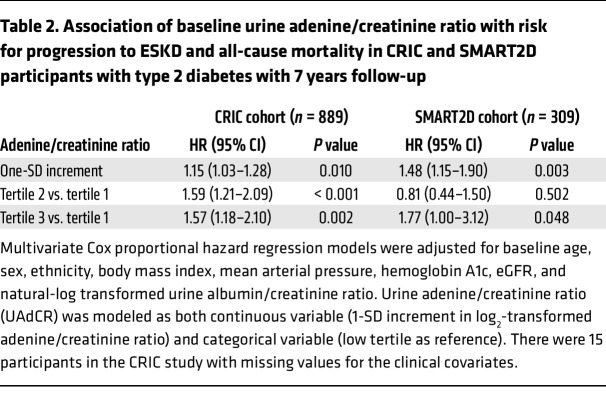
Association of baseline urine adenine/creatinine ratio with risk for progression to ESKD and all-cause mortality in CRIC and SMART2D participants with type 2 diabetes with 7 years follow-up

**Table 1:**
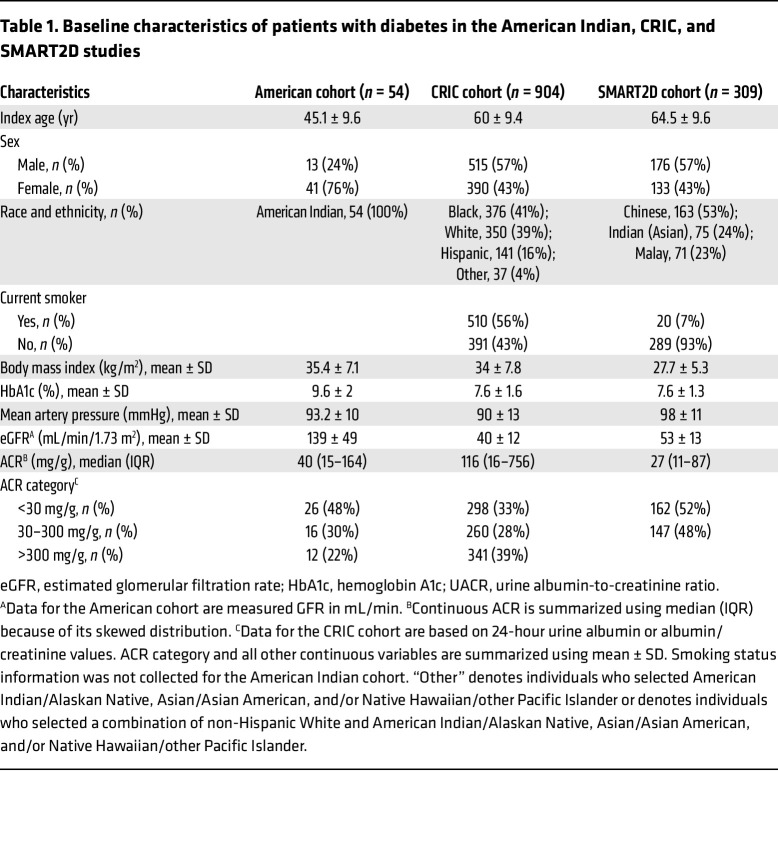
Baseline characteristics of patients with diabetes in the American Indian, CRIC, and SMART2D studies

## Version 1 08/24/2023

In-Press Preview

## Version 2 10/16/2023

Electronic publication
