# Supplementary material for: Endogenous adenine mediates kidney injury in diabetic models and predicts diabetic kidney disease in patients
Source: J Clin Invest. 2023 Oct 16;133(20):e170341. doi: 10.1172/JCI170341 (PMC10575723; doi:10.1172/JCI170341)
Supplement: Supplemental data [file jci-133-170341-s215.pdf]

Supplementary Figures and Tables

Supplementary Table 1. Hazard Ratios of ESKD or >50% decline in GFR incidence between tertiles of urine adenine/creatinine ratio (UAdCR) in American Indian cohort without macroalbuminuria.

			Continuous			T2 vs T1			T3 vs T1	
**American Indians**	**N**	**HR**	**95% CI**	**P value**	**HR**	**95% CI**	**P value**	**HR**	**95% CI**	**P value**
No macroalbuminuria	42	1.89	1.18-3.02	0.008	1.60	0.52-4.90	0.41	4.47	1.53-13.06	0.006

HR = hazard ratio; CI = confidence interval; No macroalbuminuria (ACR <300 mg/g). T1 = lowest tertile of UAdCR, T2=middle tertile of UAdCR and T3 = highest tertile of UAdCR. For the continuous measure of UAdCR, HR is per standard deviation in natural log-transformed UAdCR.

Supplementary Table 2. Association of baseline urine adenine/creatinine ratio (UAdCR) with risk for progression to ESKD in CRIC and SMART2D participants with non-macroalbuminuria and type 2 diabetes with 7 year follow up.

	**CRIC cohort (N=551)**		**SMART2D cohort (N=309)**	
**Adenine/Creatinine ratio**	**HR (95% CI)**	**p value**	**HR (95% CI)**	**p value**
1-SD increment	1.37 (1.08-1.73)	0.011	1.70 (1.21-2.39)	0.002
Tertile 2 *vs* tertile 1	1.96 (1.06-3.60)	0.031	0.91 (0.37-2.28)	0.848
Tertile 3 *vs* tertile 1	2.36 (1.26-4.39)	0.006	2.39 (1.08-5.29)	0.032

Multivariate Cox proportional hazard regression models were adjusted for baseline age, sex, ethnicity, body mass index, mean arterial pressure, hemoglobin A1c, eGFR and natural-log transformed urine albumin/creatinine ratio. UAdCR was modelled as both continuous variable (1-SD increment in log2-transformed adenine/creatinine ratio) and categorical variable (low tertile as reference). There were 7 subjects in CRIC with missing values for the clinical co-variates.

Supplement Table 3. Correlations between urine adenine/creatinine ratio (UAdCR) and urine albumin/creatinine ratio (UACR) or the estimated glomerular filtration rate (eGFR) in the non-macro groups from CRIC and SMART2D.

**Cohort**	**UACR (r, p-value)**	**eGFR (r, p-value)**
**CRIC (n=551)**	r=-0.02, *p*=0.62	r=0.01, *p*=0.74
**SMART2D (n=309)**	r=0.01, *p*=0.82	r=-0.06, *p*=0.28

Supplementary Table 4. Baseline characteristics of participants in ATIRMA, KPMP, and CROCODILE studies.

Characteristics	ATIRMA (n=40)	KPMP (n=54)	CROCODILE (n=15)
Age, years	24.3 ± 5.1	(50.5 - 59.5) ± 12.7	24.1 ± 3.9
Sex			
Male, n (%)	20 (50.0)	22 (40)	7 (46.7)
Weight, kg	71.3 ± 12.6	..	73.5 ± 12.7
BMI, kg/m^2	24.1 ± 3.2	..	25.2 ± 3.9
HRT, n (%)	..	20 (36.4)	5 (33.3)
T1D, n (%)	..	4 (7.3)	8 (51.3)
T2D, n (%)	..	30 (56.4)	2 (13.3)
HbA1C (%)	7.8 ± 0.9	..	.
MAP, mmHg	79.5 ± 7.4	..	.
eGFR*, mL/min/1.73m^2	149.8 ± 33.9	(50.6 - 59.6) ± 23.6	148.8 ± 34.6
Urine ACR (mg/g)	10.35 ± 5.93	..	7.8 ± 7.9

BMI: body-mass index, SBP: systolic blood pressure, DBP: diastolic blood pressure, MAP: mean arterial pressure, GFR: glomerular filtration rate, ERPF: estimated renal plasma flow, HbA1C: glycated hemoglobin A1c. (values expressed as Mean ± SD)

Supplementary Table 5. Correlation of urine adenine/creatinine ratio (UAdCR) with tissue levels of adenine.

Spatial Adenine	Correlation with Urine Adenine/Creatinine with Kidney Adenine by MALDI-MSI
Glom Adenine	R = 0.64, p<0.001
Non-Glom Adenine	R = 0.81, p<0.001
Whole Slide Image Adenine	R = 0.73, p<0.001

Correlation of UAdCR measurements with tissue levels of adenine by spatial metabolomics of rat kidney samples (n=9).

Supplementary Table 6. Clinical parameter in MTDIA treated db/db mice with type 2 diabets

Parameters	db/m (n=6)	db/m + MTDIA (n=6)	db/db (n=6)	db/db + MTDIA (n=6)
Body weight (g)	29.0 ±2.0	28.9 ±0.3	45.0 ±4.6	43.1 ±8.8
Blood glucose (mg/dL)	121.7 ±8.3	142.4 ±38.3	477.0 ±137.8	409.0 ±150.8
Food intake (g/mouse/day)	4.6 ±0.9	5.4 ±1.7	5.8 ±0.6	5.5 ±1.1
Water intake (g/mouse/day)	4.3 ± 0.8	4.5 ±1.0	9.7 ±3.6	8.2 ±2.7

Supplementary Table 7. Kidney metabolites in MTDIA treated db/db mice with type 2 diabets

**Metabolites** (mM/mg of protein)	**db/db** (mean ± SD)	**db/db + MTDIA** (mean ± SD)
**Alanine**	210 ±28.2	252 ±69.1
**Arginine**	25 ±5.6	29 ±9.9
**Asparagine**	54 ±12.9	64 ± 17.0
**Aspartic acid**	188 ±26.9	177 ±27.3
**Cysteine**	88 ±10.4	85 ±13.1
**Glutamic acid**	584 ±55.2	536 ±59.6
**Glutamine**	124 ±14.5	124 ±31.4
**Glycine**	472 ±38.7	514 ±82.4
**Histidine**	21 ±4.4	25 ±10.2
**Isoleucine**	25 ±4.0	35 ±13.3
**Leucine**	60 ±10.0	81 ±30.9
**Lysine**	35 ±9.0	46 ±19.3
**Methionine**	25 ±5.0	31 ±11.0
**Phenlyalanine**	27 ±6.6	28 ±8.2
**Proline**	44 ±9.9	66 ±34.8
**Serine**	96 ±21.3	125 ±48.4
**Threonine**	56 ±10.1	72 ±27.1
**Tryptophan**	6 ±1.4	8 ±2.6
**Tyrosine**	35 ±7.0	47 ±17.8
**Valine**	46 ±6.7	64 ±24.0
**3- hydroxykynurenine**	N.D.	N.D.
**Betaine**	118 ±54.5	152 ±67.0
**DL-homocysteine**	N.D.	N.D.
**GABA**	3 ±0.6	9 ±9.2
**Glycyl-histidine**	0.13 ±0.03	0.15 ±0.05
**Kynurenine**	N.D.	N.D.
**L-a-aminobutyric acid**	0.85 ±0.77	0.62 ±0.26
**Nicotinic acid**	N.D.	N.D.
**Ornithine**	5 ±1.7	11 ±7.6
**Pipecolate**	0.66 ±0.21	0.65 ±0.08
**Serotonin**	N.D.	N.D.
**Sulpiride**	N.D.	N.D.

The metabolites in the kidney were analyzed by ZipChip coupled with mass spectrometry.

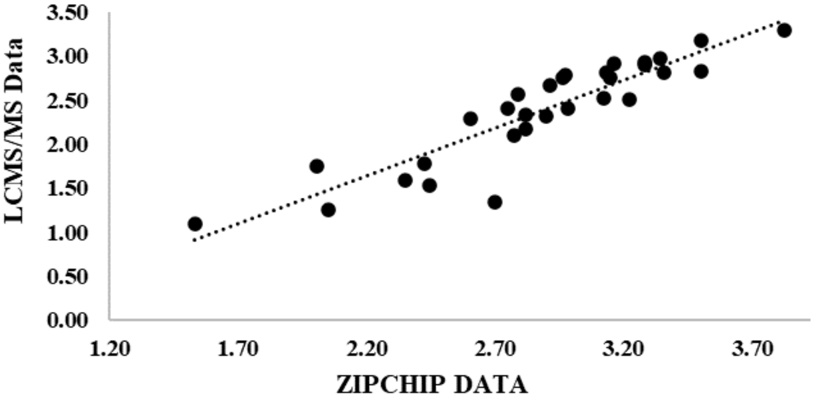

Supplementary Figure 1. ZipChip urine adenine/creatinine assay correlates with LC-MS/MS. A ZipChip urine adenine/creatinine assay was developed and found to highly correlate with urine adenine/creatinine measured by LC-MS/MS. (n=23 samples, r=0.90, p<0.0001). Raw data was log-transformed and then normalized to urine creatinine. The unit for adenine concentration in the urine is nM/mM creatinine.

A. B.

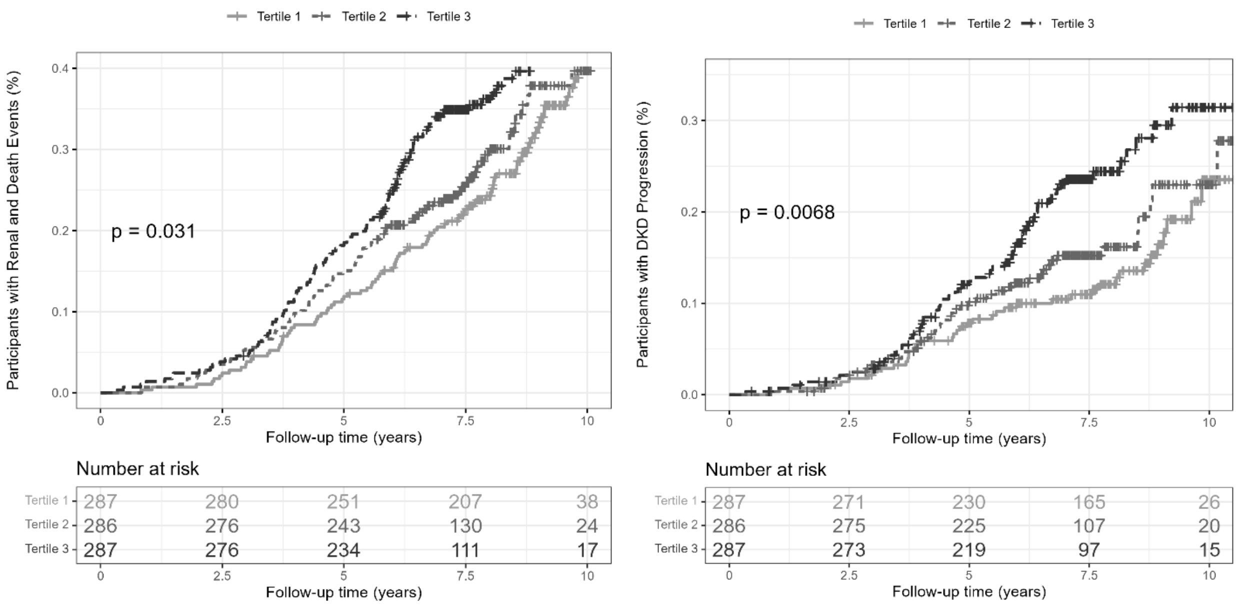

Supplementary Figure 2. High urine adenine/creatinine ratio (UAdCR) levels identify patients with diabetes who are at high risk of ESKD and mortality (A) and ESKD (B) (combined CRIC and SMART2D). Participants with diabetes in the CRIC cohort and SMART2D cohorts had UAdCR levels measured within 1 year of enrollment and followed for 8-10 years. The top tertile had the highest risk of ESKD and all cause mortality (A) and ESKD alone (B).

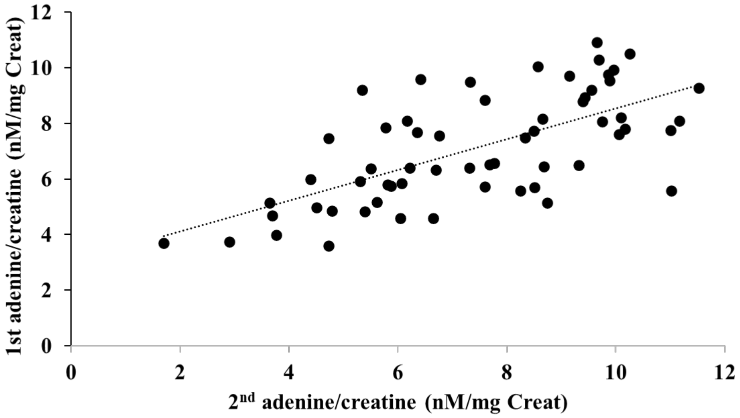

Supplementary Figure 3. Urine adenine/creatinine ratio (UAdCR) levels are stable over time in American Indians. Median time between measures of UAdCR is 12 months (IQR 11.8-12.5 months), R=0.665, p<0.0001, n=54 pairs).

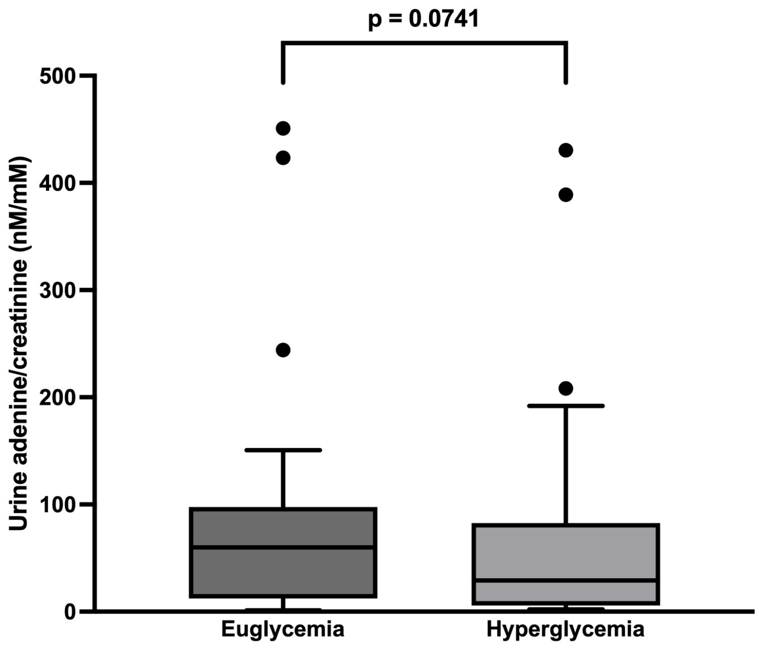

Supplementary Figure 4. Hyperglycemia does not increase urine adenine/creatinine ratio (UAdCR) levels. Patients with T1 diabetes underwent euglycemic clamp or hyperglycemic clamp with UAdCR measured at end of clamp period (n=40 patients).

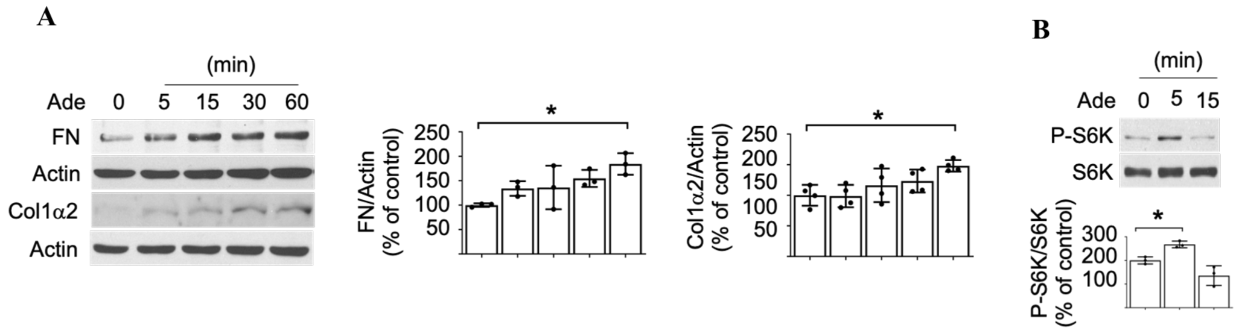

Supplementary Figure 5. Adenine stimulates fibronectin, type I collagen and mTOR in human proximal tubular epithelial cells. Adenine administration to human kidney-2 (HK2) cells increases production of fibronectin and alpha2 chain of type I collagen (A) within 1h. Adenine stimulates mTOR activity within 5m of exposure as measured by phosphorylation of S6 kinase (B) (n=3 samples/group *p<0.05).

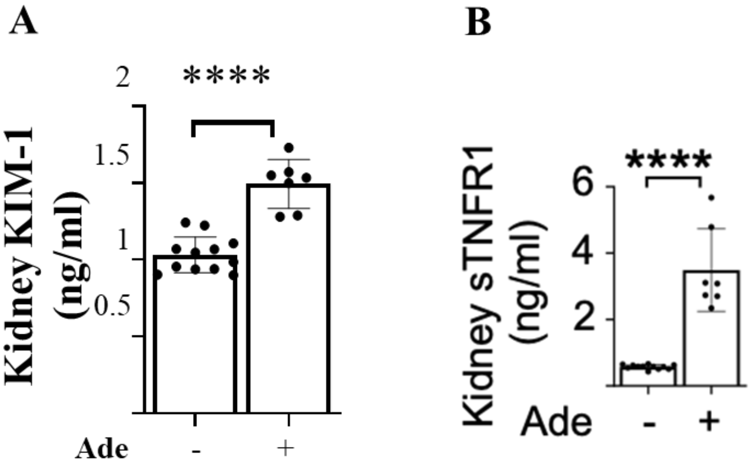

Supplementary Figure 6. Adenine stimulates kidney KIM-1 and kidney sTNFR1 levels. Adenine administration to mice increases kidney levels of KIM-1 (A) and sTNFR1 (B). (n=12 in control group and n=7 in adenine treated group, ****p<0.001).

Supplemental Acknowledgments

The author affiliations are as follows: the Center for Precision Medicine, the University of Texas Health San Antonio, TX, USA (G.Z., A.F., L.H., H.J.L., M.A.V., C.A., J.G., K.S.), the Division of Nephrology, Department of Medicine, the University of Texas Health San Antonio, TX, USA (G.Z., H.J.L., K.S.), the Department of Pharmacological Sciences and Institute for Systems Biomedicine Icahn School of Medicine at Mount Sinai, New York, NY, USA (J.H., R.I.), the Chronic Kidney Disease Section, National Institute of Diabetes and Digestive and Kidney Diseases, Phoenix, AZ (H.C.L., R.G.N.), the Clinical Research Unit, Khoo Teck Puat Hospital, Singapore (J.J.L., S.C.L., J.G.), the Department of Population Health Sciences, University of Texas Health San Antonio, 7703 Floyd Curl Drive, San Antonio, TX, 78229, USA (R.F.), the SygnaMap, San Antonio, Texas, USA (L.H.), Audie L. Murphy Memorial VA Hospital, South Texas Veterans Health Care System, San Antonio, TX, USA (G.Z., H.J.L., K.S.), the Department of Pathology, University of Texas Health San Antonio, TX, USA (M.A.V.), the Department of Family Medicine and Public Health, Herbert Werthein School of Public Health, University of California-San Diego, La Jolla, California (L.N.), the University of California-San Diego Moores Cancer Center, University of California-San Diego, La Jolla, California (L.N., J.Z.), the Center for Molecular Medicine, Vienna, Austria (V.S.), the Department of Biostatistics, Fielding School of Public Health, University of California, Los Angeles, California (B.K.), the Division of Nephrology, Department of Medicine, Boston University, Boston, MA (S.W.), the Department of Medicine, University of Washington, Seattle, WA, USA and the Division of Nephrology, Department of Medicine, Kidney Research Institute, University of Washington, Seattle, Washington, USA (J.H., K.T., B.K., I.D.B.), the Division of Nephrology, Department of Medicine and Section of Endocrinology, Department of Pediatrics, University of Colorado Anschutz Medical Campus, Aurora, CO, USA (P.B.), the Institute of Molecular Systems Biology, ETH Zurich, 8093, Zurich, Switzerland (T.F.), the Center for Clinical Epidemiology and Biostistics, Perelman School of Medicine at the University of Pennsylvania and the Department of Biostatistics, Epidemiology, and Informatics, Perelman School of Medicine at the University of Pennsylvania (H.F.), the Epigen Biosciences, Inc., San Diego, California, USA (F.C.T.), the Cleveland Clinic, Cleveland, Ohio (J.S.), Department of Clinical Pharmacy and Pharmacology, University of Groningen, University Medical Center Groningen, Groningen, the Netherlands (H.L.H.), The George Institute for Global Health, Sydney, Australia (H.L.H.), the Department of Internal Medicine, University of Michigan, Ann Arbor, Michigan, USA (R.M., E.O., J.H., M.K.), the Environmental Molecular Sciences Laboratory, Pacific Northwest National Laboratory, Richland, WA, USA (C.A.), the Structural and Computational Biology Unit, European

Molecular Biology Laboratory, Heidelberg, Germany (T.A.), the Diabetes Center, Admiralty Medical Center, Singapore and the Saw Swee Hock School of Public Health, National University of Singapore, Singapore and the Lee Kong Chian School of Medicine, Nayang Technological Univeristy, Singapore (S.C.L.)
